# User Perceptions of Wearability of Knitted Sensor Garments for Long-Term Monitoring of Breathing Health: Thematic Analysis of Focus Groups and a Questionnaire Survey

**DOI:** 10.2196/58166

**Published:** 2024-12-10

**Authors:** Kristel Fobelets, Nikita Mohanty, Mara Thielemans, Lieze Thielemans, Gillian Lake-Thompson, Meijing Liu, Kate Jopling, Kai Yang

**Affiliations:** 1 Department of Electrical and Electronic Engineering Imperial College London South Kensington United Kingdom; 2 Royal Free London NHS Foundation Trust London United Kingdom; 3 WSA E-Textile Innovation Lab Winchester School of Art University of Southampton Winchester United Kingdom

**Keywords:** health technology, wearability of knitted sensors, focus groups, asthma observation, medical device, wearable device, medical instrument, medical equipment, medical tool, sensor, physiological sensor, focus group, breathing, respiratory, respirology, lung, monitoring, monitor, health monitoring

## Abstract

**Background:**

Long-term unobtrusive monitoring of breathing patterns can potentially give a more realistic insight into the respiratory health of people with asthma or chronic obstructive pulmonary disease than brief tests performed in medical environments. However, it is uncertain whether users would be willing to wear these sensor garments long term.

**Objective:**

Our objective was to explore whether users would wear ordinary looking knitted garments with unobtrusive knitted-in breathing sensors long term to monitor their lung health and under what conditions.

**Methods:**

Multiple knitted breathing sensor garments, developed and fabricated by the research team, were presented during a demonstration. Participants were encouraged to touch and feel the garments and ask questions. This was followed by two semistructured, independently led focus groups with a total of 16 adults, of whom 4 had asthma. The focus group conversations were recorded and transcribed. Thematic analysis was carried out by three independent researchers in 3 phases consisting of familiarization with the data, independent coding, and overarching theme definition. Participants also completed a web-based questionnaire to probe opinion about wearability and functionality of the garments. Quantitative analysis of the sensors’ performance was mapped to participants’ garment preference to support the feasibility of the technology for long-term wear.

**Results:**

Key points extracted from the qualitative data were (1) garments are more likely to be worn if medically prescribed, (2) a cotton vest worn as underwear was preferred, and (3) a breathing crisis warning system was seen as a promising application. The qualitative analysis showed a preference for a loose-fitting garment style with short sleeves (13/16 participants), 11 out of 16 would also wear snug fitting garments and none of the participants would wear tight-fitting garments over a long period of time. In total, 10 out of 16 participants would wear the snug fitting knitted garment for the whole day and 13 out of 16 would be happy to wear it only during the night if not too hot. The sensitivity demands on the knitted wearable sensors can be aligned with most users’ garment preferences (snug fit).

**Conclusions:**

There is an overall positive opinion about wearing a knitted sensor garment over a long period of time for monitoring respiratory health. The knit cannot be tight but a snugly fitted vest as underwear in a breathable material is acceptable for most participants. These requirements can be fulfilled with the proposed garments. Participants with asthma supported using it as a sensor garment connected to an asthma attack alert system.

## Introduction

Smart garments are a type of wearable technology where the sensors are integrated into clothing that is being worn during day-to-day activities. The technology incorporated in the clothing provides various functions [1], such as tracking biometric data [2], controlling other smart devices [3], or enhancing comfort [4], and performance [5]. This wearable technology is different from smart watches, rings, or glasses (smart accessories) as the tech in garments sits within elastic shirts or trousers. Therefore, these garments impose higher demands on wearability and signal processing [6]. While smart accessories already have a large market share, smart garments are awaiting further investments in research and development to potentially break through into the commercial market [7]. One of the observations that can be made from the smart garment products currently available on the market is that their style relates closely with sportswear [8,9]. Commercial implementations provide insights into the user’s health and wellness, as well as personalized coaching and sports performance improvement recommendations. The application areas of commercial smart garment implementations offer real-time feedback on sleep quality [10] or sport performance [11-15] by measuring heart and respiration rate, body temperature, and body position. The marketing of smart garments for medical applications however does face challenges [16,17]. Many smart garments are not yet affordable for the mass consumer market as they use expensive fibers, fabrication processes, and technology [18]. Since this technology is new, further development in reliability is needed and regulatory approvals and certification processes are slow and expensive, forming a hurdle at the initial stages of commercialization. As a result, the main areas that are being targeted by this technology are sports and well-being wear. This might lead to the perception that smart garments are mainly for fitness rather than for the people who need wearable health devices for continuous health monitoring, such as those with cardiovascular or lung disease [19]. Thus, the style of currently available smart garments can influence consumer acceptance, especially for the older generation or those less fitness inclined. Therefore, alternative approaches toward smart wearable garments for health care and their acceptability for the user are investigated [20]. We developed a knitted breathing sensor garment [21,22] with a more classical look and feel that could potentially lead to wider user acceptance and market adoption, especially in areas related to long-term breathing monitoring.

Long-term breathing monitoring has been identified as a method to help people with lung diseases such as asthma [23,24]. Exploring the preferences of users is an important ingredient in developing wearable sensor garments for long-term use [25]. The ability to measure and collect breathing data using our knitted sensors was established before [21,22]. This paper aims to explore user perceptions of our knitted breathing sensor garment to assess how it may evolve to meet user requirements for long-term breathing monitoring. We then investigate whether the preferred sensor implementation can also output good quality breathing signals. With the aim to map compatibility of user requirements to sensor performance, we report on quality of the knitted sensors in terms of sensitivity to chest and abdomen circumference variations during simulated breathing using an in-house made chest phantom.

## Methods

### Recruitment

A standard e-mail was sent to final year engineering students, research fellows and support staff in the Electrical Engineering Department of Imperial College London and encouraged the engagement of associated contacts. A total of 16 participants were recruited (Table 1). Written and verbal consent was obtained from all participants. A questionnaire was designed in Qualtrics XM under Imperial College London license. The protocol for this study was approved by the Science, Engineering and Technology Research Ethics Committee of Imperial College London (application ID 6620621). Participants were only asked to report on whether they were diagnosed with asthma, as the garments were specifically designed to monitor breathing health.

**Table 1:** Demographics of participants.

Demographic		Participants, n (%)	
**Sex**			
	Male		9 (56)
	Female		7 (44)
**Age (years)**			
	18-30		10 (62)
	31-50		3 (19)
	>50		3 (19)
**Asthma diagnosis**			
	Yes		4 (25)
	No		12 (75)
**Background**			
	Researcher		8 (50)
	Health Care Professional		2 (12.5)
	No formal research training		6 (37.5)

### Methodology

**Figure 1:**
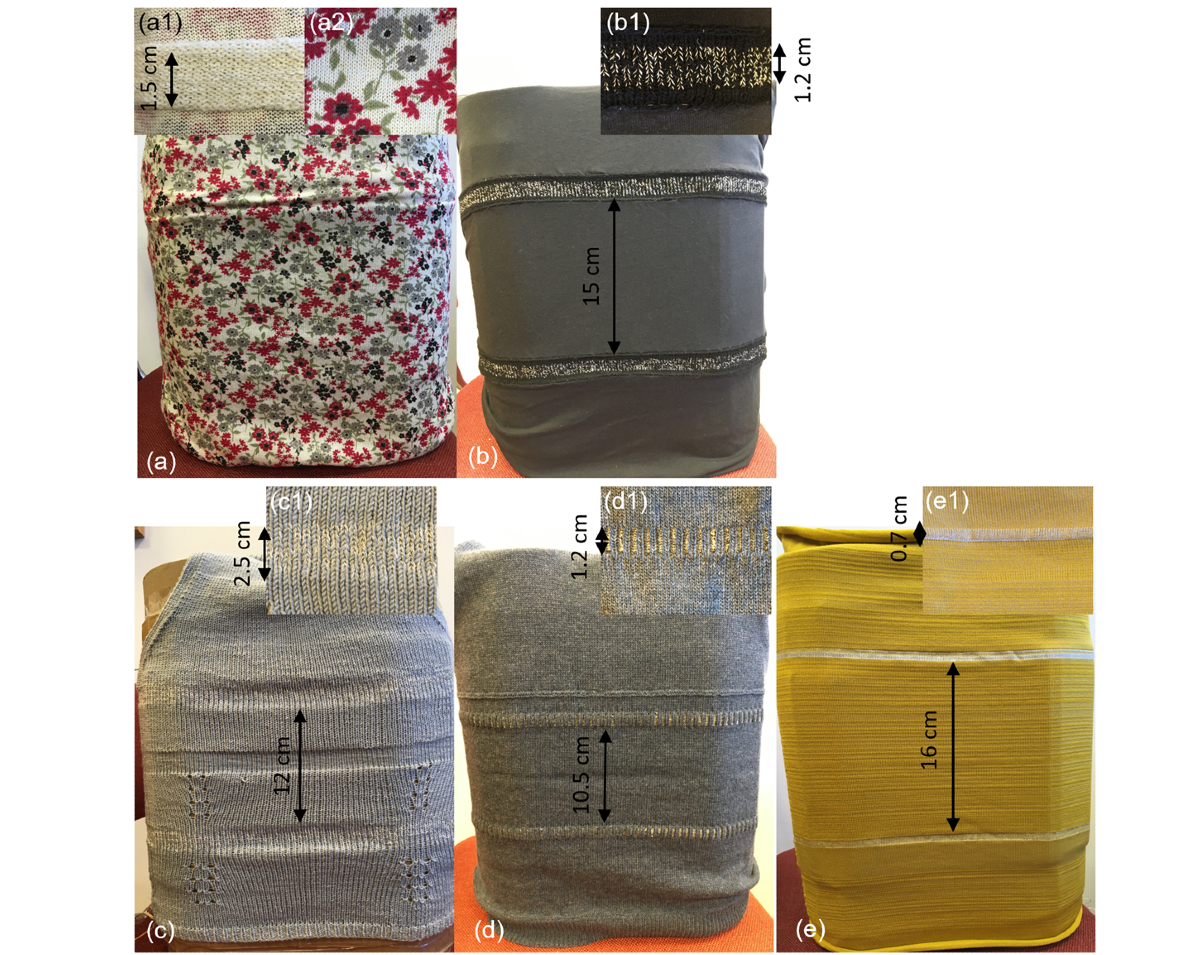
Pictures of the different implementations of the knitted sensor garment. The inset zooms in on the sensors. Top row: modular systems with knitted sensors are added to a garment. Bottom row: fully knitted implementations.

**Figure 2:**
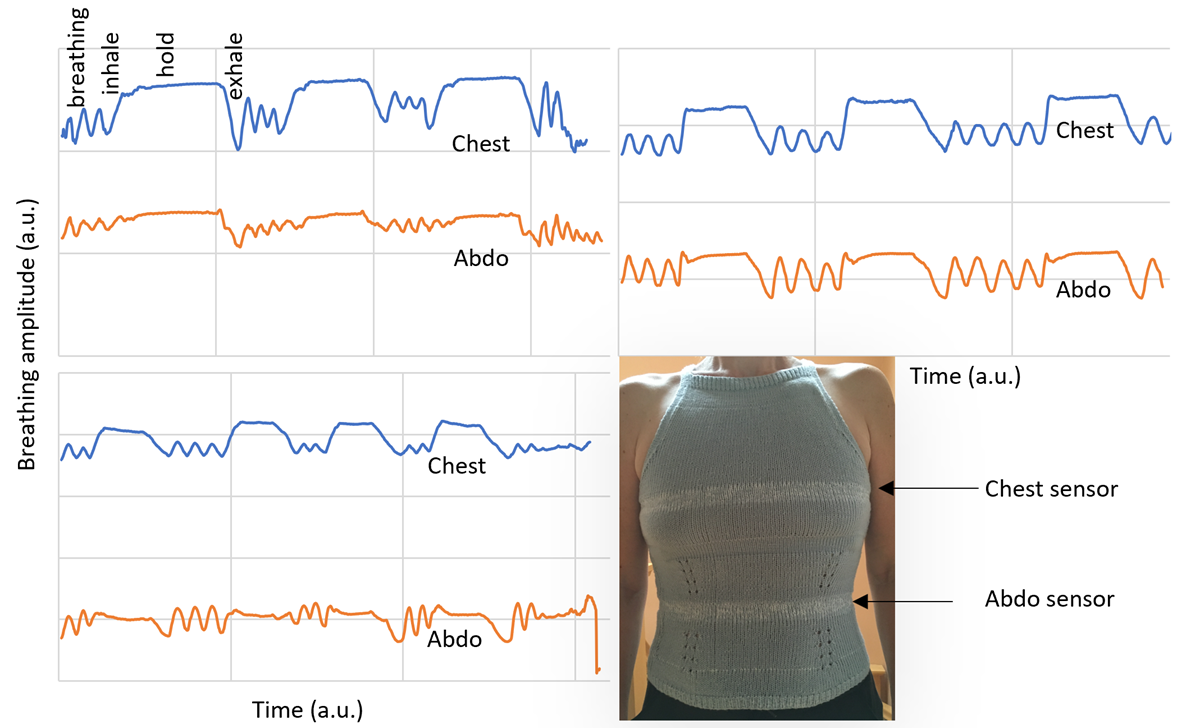
The breathing patterns–breathing amplitude variations as a function of time for 3 different volunteers using an aerosol inhaler technique and wearing the tank top sensor garment shown bottom right. This sensor garment is hand knitted and has 2 sensors, one at the chest and one at the abdomen as indicated by the arrows.

Different knitted sensor garments were introduced by the lead investigator [KF] (pictures are presented in Figure 1). These garments were designed and created by the research team with a variety of knitting techniques and materials, including modular sensor implementations in cotton and normal fit (Figures 1A and 1B) given a hidden and visible implementation, respectively, fully handknitted snug fit in microfibra (92%) and elastane (8%, Figure 1C) and 2 machine knitted implementations, 1 snug fit in wool using a Kniterate tabletop circular knitting machine by Ecoknitware, United Kingdom (Figure 1D) and 1 tight fit using Fluid (2% elastane, 7% nylon, and 91% viscose) combined with an elastomeric yarn (19% Lycra and 81% nylon) for the bodice and a conductive yarn (Elektrisola yarn 80% silver and 20% copper) combined with the same elastomeric yarn for the sensors using a 12 gauge Shima Seiki knitting machine at the Winchester School of Art, United Kingdom (Figure 1E). Participants were encouraged to touch and feel the garments during the presentation and subsequent focus groups.

The theme of knitted garments and their sensor capabilities was introduced at the start of the focus groups using a PowerPoint presentation. A total of 2 focus groups were set up with 8 participants in each. Each focus group had an equal gender ratio. Each focus group was led by an independent facilitator. The discussion topics for the focus group were predetermined to facilitate and guide conversations and give some structure but focus group leaders allowed participants to go beyond these questions. The focus group topics were the following:

1. Probe the participants’ impressions of the wearability of the knitted garments that were demonstrated.
2. Breach the topic of the necessity for electronic readout in smart garments and probe acceptance.
3. Probe the opinion of the participants on the access to the data and their use.
4. Allow participants to voice concerns and contribute ideas.

The participants also completed a survey concerning their personal opinion on the wearability of the knitted garments presented immediately after the focus groups. The survey was hosted by Qualtrics.

### Data Analysis

Transcripts were made by [NM] from both recordings after the meeting and fully anonymized for the analysis. Thematic analysis [26] was used to identify, analyze and report themes within the qualitative data contained in the transcripts of the focus groups. It involved 6 phases that were carried out by a set of 3 independent researchers. The phases were as follows: familiarization with the data (all researchers involved KF, NM, and MT), generating initial codes (2 involved KF and NM), searching for themes (2 involved KT and NM), reviewing themes (specialist involved MT), defining and naming themes (specialist involved MT), and producing the report (all researchers involved KF, NM, MT, and LT). Qualtrics was used for the statistical analysis of the questionnaire.

The breathing sensors’ operational characteristics were determined by using a specially designed rib phantom that simulates breathing and home-built battery powered readout electronics [21]. The sensitivity of each sensor was extracted from the measurements that relate the changes in readout output to the changes in circumference of the rib phantom. The sensors were snug fitted on the rib phantom except for the tight fit design of Figure 1E.

## Results

### Research Characteristics

The focus group facilitators were both female, 1 with an MSc in Analogue and Digital Integrated Circuit Design (ADIC) and the other, a junior doctor in General Practice training. The survey was created and conducted by an MSc in psychology, the MSc in ADIC and a professor of electrical engineering.

### Thematic Analysis

Based on the coding, 3 overarching themes with each 2-3 additional subthemes were identified and are given in Textbox 1 with some specific representative coding examples.

**Three overarching themes each with 2-3 subthemes identified from the coding of the transcripts of the 2 focus group discussions. Some specific coding is added for each subtheme.**

**Everyday usage of garment**

- Wearability of the garment
  Underwear in cotton.
  Feeling of coils against the skin.
  More “giveable” fabric.
- Maintenance of the garment
  Can it be washed?
  Longevity.
  Waterproof electronics.
- Placement and comfort of the electronics
  Not a big box.
  Hidden.
  Pocket.

**Intention for the use of the garment**

- Commercial vs health
  Visual appeal or style.
  Prescribed by general medical practitioner.
  Illness connotation.
- Cost of garment
  For health, not expensive.
  For athletes “high end stuff.”
  Needs-based.
- How the garment could be used
  Reduce hospital admission.
  Caught off guard.
  Second clearance.

**Data output**

- How the data are used
  Warning or alert.
  Training.
  Trend.
- Awareness of wearing the garment
  Overwhelming.
  Conscious.
  Influence breathing.

#### Findings on Everyday Garment Usage

The focus group on everyday usage of the garment revealed some insights and preferences of potential customers. Participants expressed that they would like the style and materials of the garment to be appropriate for different seasons:

I was looking at all the knits and they seemed like they’d keep me so warm. I wouldn’t be able to wear them in summer

and proposed a cotton underwear implementation that would not be subject to specific style requirements:

If you want this to fit into all sorts of all kinds of clothing then of course it is difficult. But if they're just underwear then they can be simple just one style cotton and that's it.

They also preferred a more relaxed knit that is not rigid and tight, and easy to slip on:

I probably wouldn't wear the one that was rigid because I’d be worried that it actually changes my breathing pattern.

In this they found the garment of Figure 1D to be particularly suitable. They were concerned about the sensor wires that would be embedded in the garment, and they wanted them to have no skin contact, and to be smooth and hidden:

It would be better if it wouldn’t press against the skin directly. I’d prefer if it is disguised in order to wear them on a regular basis.

Comfort related to textiles used in clothing has been and is still a major aspect in garment design. The traditional techniques used should still be applied for smart garments [27]. Participants suggested two possible implementations: a modular garment that allows them to detach the sensors and add them to other garments:

I can bring my own top and then yeah, put the wire… So, you don't have repeated cost of wire, but just the cost of doing it.

or the sensor implementation of Figure 1E that integrates them smoothly and seamlessly into the fabric:

…the coil is more like hidden and smoother in the yellow garment.

They also wanted the hardware to be small, thin, hidden, and waterproof, and suggested that a purpose-built integrated circuit could be placed in a pocket:

You could get them on a chip and provide a waterproof coating

Finally, they emphasized that longevity is important for them:

For me personally, if I was spending that much money that would have to last me years

and they wanted the sensor wire insulation properties to be durable and wear and tear resistant:

Over time, there would be wear and tear of the garment. Would that influence the working of the coils?

#### Opinions on the Use of Knitted Sensors Garments

The participants indicated that they would be more willing to wear sensor garments if they had a medical condition that required monitoring or if their doctor recommended it to them. They did not care much about the cost or the style of the garments if this was the case:

Personally, I probably won't get this if I walk into a shop. Yeah, but if it suggested by a doctor, I could just ignore the appearance.

The participants expressed doubts about the feasibility of knitted sensor garments as a commercial product for healthy people. They cited issues such as style, aesthetics, and cost:

If it wasn't prescribed by a healthcare professional and is just commercial, the material and cost matter.

The importance of aesthetics in smart garments is a recurring theme in literature, as, for example, directly captured in the title of [28]. The participants acknowledged the potential benefits of sensor garments as a medical device, especially for preventing or detecting health problems that could lead to hospitalization:

From a paramedic point of view, I think this product could really help people learn inhaling techniques… Visualizing the patterns can assist them in training.

They suggested that sensor garments could improve their quality of life and reduce health care costs by reduced visits to the GP (general practitioner) or hospital.

#### Opinions on Smart Output Data

There were divided opinions about what part of the biosignals should be made available. Some participants preferred to have access to the raw data, while others were satisfied with the summary statistics. The reasons for preferring the raw data included curiosity and personal research:

…Various data that is stored over time can then be used to identify certain patterns or stages to look out for… and figure situations personal to the consumer.

The reasons for preferring the metrics included simplicity, convenience, and avoiding information overload:

I think that you can get obsessed if you have access to the signals… Especially for people suffering with respiratory problems like asthma for example, it might be triggering.

All participants agreed that notifications were a must feature for smart garments, especially for critical situations such as an imminent asthma attack. Notifications were also seen as useful for reminders, trends and feedback on progress:

General pop ups and an alert if something is not in order.

Participants expressed interest in a feature that would provide visual guidance on how to inhale properly to help them improve their breathing quality:

Provide general advice on good breathing practices and certain exercises to follow. It could also give more specific instructions based on your patterns and thresholds that are personalized to the consumer.

Product functionality is a major aspect in the interest of users and can drive the uptake of smart wearables if other aspects concerning wearability, comfort and aesthetics are fulfilled [29]. The participants input on the need for machine learning to improve the accuracy and personalization of the product might be mainly triggered by the majority of the participants being involved in some type of research:

Something like models to detect abnormal behavior or stuff, but following this is the customized problem that is that the data is different for individuals… it might not necessarily work for the new customer

Of note was that recyclability and the ecologically beneficial implementation of the knitted garment (which is the aim of the company Ecoknitware) was not a priority for the participants, with a lack of understanding of the material and waste issues related to fashion:

Umm, I’m not very familiar with the material.

One participant mentioned recyclability but seemed unsure of the implementation of this:

“*So you could have some sort of recycling set up by which people send you the stuff, and maybe I don't know whether you can recycle that or reuse it*.” There was more focus on style and aesthetics than sustainability: “*But if it is from a commercial brand, it’s better to visually appeal*.” “*Bit more trendy maybe*.”

Another observation is that although the facilitators of the two focus groups had different academic background knowledge, the overall themes and conclusions of both groups were similar.

Overall, the main points extracted from the qualitative data of the focus groups were as follows:

1. garments would be more likely to be worn if prescribed by a GP to be used as a medical device for health reasons.
2. due to specific style requirements on outerwear, a cotton underwear (vest) implementation was preferred.
3. garments must be comfortable, meaning smooth sensors and small electronics, and relatively easy to maintain.
4. the collected data needs to be accessible and easy to read. A warning system of imminent breathing crisis was seen as the most promising application.

### Survey Analysis

The survey covered three overarching themes: (1) whether the participants like to wear knits for everyday use, (2) whether participants preferred a certain knitted style within a given selection, and (3) whether participants would wear a knit to obtain health related information.

Each theme was split into multiple questions, probing specific items within each theme. For instance, on the theme of whether a participant would wear knitted garments for everyday use, subquestions probed for the reasons such as aesthetics, need for thermal insulation and wearing comfort. A summary of the results of the questionnaire is given in Table 2.

The results in Table 2 show that there is a general perception of knits being an acceptable fabric for everyday wear (12/16). However, the results show that personal preference for style can have a big impact on whether a knit is worn or not (wear likeliness increases from 63% to 81% if users can choose style).

Table 2 shows that a loose-fitting style is preferred (81%), closely followed by a snug fit (69%). However, a tightly fitted garment would not be worn (0%). Currently, garments with integrated sensors that measure breathing patterns require a tight fit. From a technical standpoint, this is essential to obtain high-quality signals during normal use and to minimize motion artifacts [30]. Our findings align with the challenges faced by the wearable sensor garment market. The technical necessity for a tight fit has only seen some success primarily in the sports sector. However, our knitted breathing sensor garments do not require tight fits and perform well with a snug fit during gentle ambulation and could thus be considered outside the sports sector [21]. Also of interest is that night wear would be an option if the style and material is right.

Overall, most participants are happy to wear knitted clothes because they look and feel nice. There was a 50/50 division in opinion on the thermal insulation characteristics of knits. This was also reflected in the concerns mentioned in the focus group that knits might be too warm to wear. Therefore, the material and thickness of the yarns used in the knit should be such that the knit can be worn in all seasons.

**Table 2:** Result of the questionnaire. The “strongly agree” and “agree” or the “very likely” to “likely” options are summed in the results row. This gives positive feedback on the questions only. The neutral option is not counted.

Question and responses				Participants, n (%)
**Like wearing knits?**				
	You like wearing knitted clothes			12 (75)
	You wear knitted clothes only to keep you warm			8 (50)
	You wear knitted clothes because they look nice			11 (69)
	You wear knitted clothes because they feel nice			12 (75)
**Style/** **yarn**				
	Do you like the fabric of a knitted top that was shown?			10 (63)
	**Which of the following garment styles would you wear?**			
		Tank top	4 (25)	
		Short sleeved T-shirt	10 (63)	
		Long sleeved jumper	7 (44)	
		Tight fit	0 (0)	
		Snug fit	11 (69)	
		Loose fit	13 (81)	
		Baggy fit	2 (13)	
	How likely is it that you would wear a knit in that fabric but in a style of your choice?			13 (81)
	**If you answered likely or very likely to the previous question, when would you wear the clothing? (n=13)**			
		For the whole day	8 (62)	
		Only during the night	2 (15)	
		During the night if knit in pajama style	11 (81)	
		Only for breathing training	2 (15)	
		Only for testing by breathing pattern	1 (8)	
**Health related information (n=15)**				
	How likely is it that you would look at this information to get feedback on your breathing technique?			14 (93)
	How likely would you use the information for inhaler use feedback?			13 (87)
	Would you use it to train inhaler technique?			12 (80)

### Sensor Characteristics

Figure 2 shows the breathing patterns recorded with a knitted sensor garment, either the tank top (Figure 1C, that is also shown in Figure 2, bottom right) or the hybrid design (Figure 1B). The breathing pattern is consistent with the use of an aerosol inhaler: 3 normal breaths, a sharp inhalation, and a period of holding breath, followed by a slow exhalation. This pattern is repeated 2 more times. The 3 measurements shown in Figure 2 are for 3 different female volunteers with different body shapes. The blue line shows the variation in the circumference of the chest and the orange line that of the abdomen, during the breathing exercise. They are recorded simultaneously.

Figure 2 shows the breathing pattern clearly in all 3 cases even though there are variations in body shape between the different volunteers and 2 different garment implementations were used. There are variations in the relative volume changes of the abdomen and chest sensor during breathing due to the different natural breathing method of each individual volunteer.

Although the focus groups examined user preferences for wearability, it is essential to align these preferences with the sensors’ technical capabilities. If the sensors garment’s performance doesn’t match the preferred garment design, the sensor garment will not be effective.

To evaluate the performance of the different implementations shown in Figure 1 and which were discussed during the focus groups, dynamic and static measurements of the response of these garments were carried out on a home-built rib phantom that simulates breathing patterns. The sensitivity of the sensors together with key sensor implementation characteristics is given in Table 3. All garments had the same knitted sensor implementation—10 consecutive knitted rows with Elektrisola yarn—80% silver and 20% copper. The diameter of the garments was approximately the same by adapting the number of stitches in one row to the gauge of the yarn used.

The snug fitting garments that excluded float stitches in the sensor implementation performed best with the highest sensitivity, independent on specific implementation. This is a positive result as this allows flexibility in design to users’ taste and the participants did not like the float stitch implementation which can thus be avoided in future designs. The tight fitted design did not perform as well as the others due to its mechanical resistance against change. This is also a positive result as tight fits were unpopular with the participants, although they liked the smoothness of the sensors implementation in this machine knitted approach. This smoothness in sensor implementation is more related to the small size of the stitches than the tightness of the fit and can thus be implemented in future designs with a snug rather than a tight fit.

Since the characteristics of fully knitted implementations and that of a modular design are similar, the option of both implementations remains available and can be decided in view of cost of implementation and personalized style preference.

**Table 3:** The performance parameters of the different knitted breathing sensors and some key characteristics. Garment types are as defined in Figure 1.

Garment implementation	Metal wire float stitches^a^	Stitch size (mm)	Fit	Sensitivity (kHz/cm)
Modular with hidden sensor	0	2.5	snug	7.83
Modular with visible sensors	0	2.5	snug	7.88
Full hand knit	0	3	snug	7.83
Full machine knit^a^ wool implementation	2 knit/2 float	2	snug	7.01
Full machine knit^a^ cotton implementation	2 knit/2 float	2	snug	7.01
Full machine knit–tight fit knit	0	1	tight	6.03

^a^The insulated metal wire in the sensor is not included in all stitches in these implementations. The sensor make us is 2 stitches knit with yarn and wire, followed by 2 stitches without the metal wire guiding the unstitched metal wire at the back of the garment. These are called float stitches.

## Discussions

Thematic analysis on the qualitative data of 2 focus groups and quantitative analysis based on a survey, have highlighted an inclination amongst the participants to wear the knitted sensor garment during the day or the night for medical purposes. They did not see the garment as a commercial fashion garment nor as a lifestyle statement. For long time medical wear, the participants suggested underwear (vest) in the style of a short-sleeved T-shirt in cotton that sits snug but not tight to the body and that is made in a material suitable for all seasons. The wearer should be unaware of the sensors and the readout electronics. For ease of maintenance, the participants preferred not to have to remove the electronics for laundering. Longevity of the garment is essential while price would not be a main decision factor if prescribed by a medical professional for health benefits. The participants saw a warning or alert system that is triggered when a respiratory attack is imminent as essential for the medical use of the garment. What was not explored during the focus groups was any requirement for teaching people how to wear and use the garment, especially if used in a medical setting. This is an important topic for further discussion when creating a unique specification for the garments. Focus group participants could have been asked to wear the garments themselves for their opinion on this aspect. Further research into the perceptions of people living with asthma or chronic obstructive pulmonary disease would add to the gathered data and support the development of a more targeted product suitable for long-term wear.

The technical response of the garment showed excellent performance for the preferred design supporting the feasibility of this wearable breathing sensing garment for personal health monitoring. A power supply challenge exists for all smart wearables especially in view of participants’ desire not to have to remove any electronics for laundering.

Overall, there is a strong positive opinion about wearing a cotton knit with smooth sensors over a long period of time for health monitoring and training in healthy breathing techniques. The yarn material must be appropriate for all seasons. A use case could be made in relationship to an alert system to warn the user before a respiratory attack occurs.

While a large proportion of the participants in this work have a research background that might cause bias in the qualitative data, our findings are consistent with the results of previous studies [16,18,20,27-29] relating to the acceptability of smart garments for health monitoring. This similarity suggests a common trend in user perception largely independent of the garment under study.

## Abbreviations

ADIC: Analogue and Digital Integrated Circuit Design
GP: general practitioner
